# Effectiveness of Step Goal Personalization Strategies on Physical Activity in a Mobile Health App: A Field Study

**DOI:** 10.2196/81779

**Published:** 2026-02-18

**Authors:** Xia Liu, Tammo H A Bijmolt, Marijke C Leliveld, Ernst H Noppers

**Affiliations:** 1 Department of Marketing, Faculty of Business Administration, Hebei University of Economics and Business, Xuefu Road 47, Shijiazhuang, 050061, China, 86 187 3316 3263; 2 Research Centre for Science Technology and Innovation Policy, Hebei University of Economics and Business, Shijiazhuang, China; 3 Department of Marketing, Faculty of Economics and Business, University of Groningen, Groningen, The Netherlands; 4 Menzis, Groningen, The Netherlands

**Keywords:** mobile health app, goal management, goal personalization, physical activity, difference-in-difference, propensity score matching

## Abstract

**Background:**

Goal personalization features integrated into mobile health apps have the potential to enhance physical activity, as some evidence shows that the personalized goals generated by algorithms are more effective than default or fixed goals. However, it remains unclear whether goals set by users are more effective than fixed goals and which personalization strategy is more effective for different user segments.

**Objective:**

This field study aimed to evaluate (1) the efficacy of 2 step goal personalization strategies—personalized-by-you and personalized-by-the-algorithm—and (2) which strategy is more effective among users with different activity levels.

**Methods:**

All users of SamenGezond, a Dutch mobile health app, have a default goal of 2000 steps per day, 5 days a week. For this study, 2 random groups were selected, totaling 5800 users. Subsequently, an email was sent to 3800 users in group 1, asking whether they were satisfied with their current goal. Those who were not satisfied were offered 2 personalization options: to set a goal themselves or to have the algorithm integrated in the app set goals for them. In total, 1399 users responded: 230 chose to set their own goals (personalized-by-you group), 236 opted for setting the goal by the algorithm (personalized-by-the-algorithm group), and 933 chose to keep the default goal (not-changed group). The algorithm used a moving-window percentile rank method based on step data from the previous 4 weeks. Users who did not personalize retained the default goal. The remaining 2000 users in group 2 did not receive the email and also retained the default goal. To evaluate the effectiveness of step goal personalization strategies, we used propensity score matching and difference-in-difference analysis.

**Results:**

Users in the personalized-by-you group increased weekly step count by 3793 a week, while those in the personalized-by-the-algorithm group increased by 4315 steps a week, compared with the not-changed group (users with default goals). The 2 strategies appear to have a similar effect. Interestingly, users in the not-changed group also increased their weekly steps by 1759. Furthermore, the effectiveness of each strategy varied by baseline activity level. The personalized-by-you strategy was effective for medium- (increase of 5842 steps) and high-active users (increase of 4266 steps) but not for low-active users (increase of 384 steps; *P*=.82). Conversely, the personalized-by-the-algorithm strategy was effective for low- (increase of 5095 steps) and medium-active users (increase of 5278 steps) but not for high-active users (increase of 1446 steps; *P*=.51).

**Conclusions:**

Step goal personalization demonstrates short-term effectiveness. However, their impact varies by users’ baseline activity levels, indicating the need for a tailored approach for different user segments. Future studies should examine the long-term effects of such interventions to design sustainable health behavior change strategies.

## Introduction

### Background

Despite the compelling evidence of its benefits, many barriers (eg, inconvenience, lack of time, and motivation) hinder individuals from regularly exercising. According to the World Health Organization, 1 in 4 people worldwide is physically inactive [1]. Overcoming these obstacles requires stronger motivation and self-regulation. Mobile health (mHealth) technologies offer significant potential as a solution to physical inactivity [2-4]. mHealth refers to the integration of mobile computing, medical sensors, and communication technologies, designed to deliver health care services [5]. These tools allow individuals to manage goals, track physical activity, and receive personalized feedback—making exercise more accessible and convenient. However, measurement capabilities and personalized feedback alone may not be sufficient to sustain motivation. In some cases, they can even backfire, leading to demotivation if users feel pressured or coerced [6-8].

### Goal Management and Goal Setting

This study focuses on goal management, a central feature in most mHealth apps, as goals are fundamental drivers of motivation and self-regulation [9]. Goal setting is one of the most frequently used and effective strategies for behavioral change [10,11]. Despite its demonstrated importance, an essential question remains: what kind of goals should be set to effectively motivate behavioral change?

This question sparked abundant research and theories on goal setting. For instance, one line of research recommends setting achievable and realistic goals as formalized in the SMART (specific, measurable, achievable, realistic, time-bound) framework [12,13]. Other research emphasizes the motivational benefits of challenging goals [14,15]. Despite that both approaches have merits, the majority of research on goal setting agrees that challenging goals may be discouraging if perceived as unattainable, while easy goals may fail to motivate people [11,14]. The effectiveness of goal setting thus depends critically on aligning goals with users’ abilities, needs, and contexts [11]. This raises an essential question: how can such goals be designed and calibrated effectively?

### Goal Personalization

To achieve this alignment, many mHealth apps have introduced goal personalization features. These features allow users to modify the default goal, uniform across all users, offering options to personalize the goal to better align with individual circumstances. The process of goal personalization can typically take two forms: (1) personalized-by-you (also known as “customization”), where users set their own goals, and (2) personalized-by-the-algorithm, where the app generates goals based on users’ previous exercise data with an integrated algorithm [16].

Goal personalization is expected to increase physical activity compared to no personalization (with default goals) for a few reasons. First, from a goal-setting theory perspective, personalized goals—whether self-set or algorithm-generated—might be more effective than default goals because they accommodate individual abilities and situational context. In addition, from the self-regulation perspective, goal personalization may have the potential to increase physical activity compared to default goals, since the process of personalization encourages active user engagement. This may further foster a greater sense of control and autonomy [17] and reinforce users’ perception of themselves as the originators of their goals [18-20]. As a result, users may experience less depletion of self-control resources and feel more energized and maintain greater commitment to achieving their goals [21]. Previous research has partly demonstrated the efficacy of step goal personalization. For example, a study found that a personalized-by-the-algorithm strategy led users to take more steps than a static and default goal (eg, 10,000 steps) [22]. Similarly, 2 more studies reported that the personalized goals generated by the algorithm led to a slower decline in step counts over time compared to nonpersonalized, static goals [23,24].

Despite these promising findings, existing research faces several limitations. First, most studies compare the effectiveness of goals personalized by the algorithm with high, fixed goals (eg, 10,000 steps), which may be challenging for most users [22-24]. It remains unclear whether personalization is still effective when compared to low default goals (eg, 2000 steps). Moreover, much of the prior research on the effect of personalized-by-the-algorithm relies on small-scale randomized controlled trials, often involving fewer than 50 participants per condition, limiting the generalizability of findings [22-24]. For example, one study stated in its limitation section a low sample size of 64 staff workers from a certain university with a dominant proportion of female participants (83%). The results may not generalize to the general public [23]. Second, as far as we know, no previous research has examined the effectiveness of the personalized-by-you approach, relative to existing default goals, in promoting physical activity, underscoring the need for further research. Third, and more importantly, no evidence exists on which personalization strategy is more effective for different types of users. The goal-setting literature offers mixed insights: some studies suggest that self-set goals may be less effective than the assigned goals due to the sustained effort required from users [23,25,26], while others argue that self-set goals promote greater autonomy and may thus be more motivating [27,28]. It is possible that the effectiveness of goal-setting approaches is moderated by other factors, such as individuals’ locus of control [29] or their levels of ability [11]. Thus, it is important to investigate which goal personalization strategy is more effective across different types of users.

Collectively, these limitations underscore the need to examine the effects of both goal personalization strategies and investigate which goal personalization strategy works best for different types of users.

### Objectives

To address these gaps, this study investigates whether goal personalization increases health behavior and how the different routes of setting the personalized goal affect health behavior. Specifically, this study investigates the effect of goal personalization on physical activity by examining whether 2 personalization strategies (personalized-by-you and personalized-by-the-algorithm) outperform no personalization (with uniform default goals). Additionally, it also examines which strategy is more effective among users with different baseline activity levels (ie, high-, medium-, and low-active users), providing a more comprehensive understanding of how goal personalization operates in real-world settings.

## Methods

### Ethical Considerations

This study utilized data from an experiment implemented by SamenGezond, which was designed to optimize the effectiveness of the SamenGezond program. Specifically, this research involved a secondary analysis of the data from adult users of the SamenGezond, an mHealth app, collected by SamenGezond in accordance with its terms of use and privacy policy. The policy states that SamenGezond measures and stores user activities and analyzes them (1) to provide feedback and advice and (2) to test and optimize the effectiveness of SamenGezond. By agreeing to the privacy policy, users consented to the use of their data for analytical purposes. All data used in this research were fully deidentified and aggregated. The use of these data was approved by the institutional research board of the Faculty of Economics and Business of the University of Groningen (approval number FEB-20250424‐01512).

### Study Design

**Figure 1.**
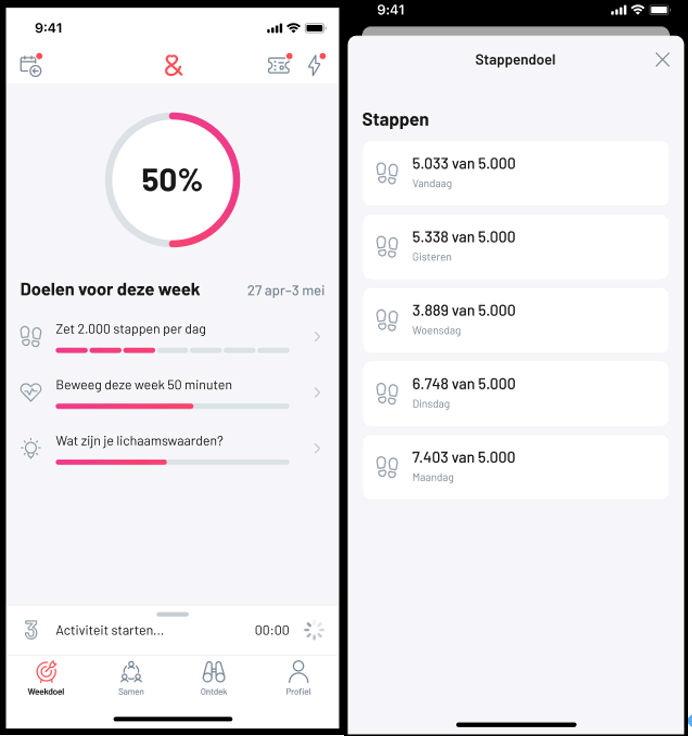
Screenshots of the (Dutch language) mobile health app.

SamenGezond (which translates to “healthy together”) is a health app based in the Netherlands, launched in October 2017. Similar to other mHealth apps, it aims to enhance users’ health through features such as goal management, GPS-enabled physical activity tracking, and personalized feedback. In addition, the app offers access to health-related papers, expert advice, support from a web-based coach, and opportunities to join exercise communities. Each user is initially assigned a default step goal of 2000 steps per day, 5 days a week. As shown in Figure 1, the app’s main page (left panel) displays a circular progress indicator representing the percentage of the user’s weekly goal achieved. The circle is color-coded according to the completion level. Below this indicator, the user’s step goal is shown, translated as “Take 2000 steps per day,” followed by a progress bar indicating weekly performance. Users can also view their total number of steps for each day and the step goal for the week on a separate interface (right panel of Figure 1).

To obtain a valid sample for the study, we used 3 criteria to filter the users in the database. The criteria were, first, that the user had to be a member of the app for at least 4 weeks to ensure some experience with the app; otherwise, the participants would not be able to answer questions on their experience with the step goal. Second, the user must have used this app in the last year because historical exercise data are required to generate a new step goal for users. Third, the users must have subscribed to email communication to ensure the survey can be sent. After applying these criteria, we used the R package dplyr to randomly choose users in the database, ensuring that each user had an equal probability of being included in the sample. Specifically, we used the sample_frac function to select 10% of the users for group 1 and the sample_n function to select users for group 2. As a result, 3800 users were chosen for group 1 and 2000 users were chosen for group 2.

**Figure 2.**
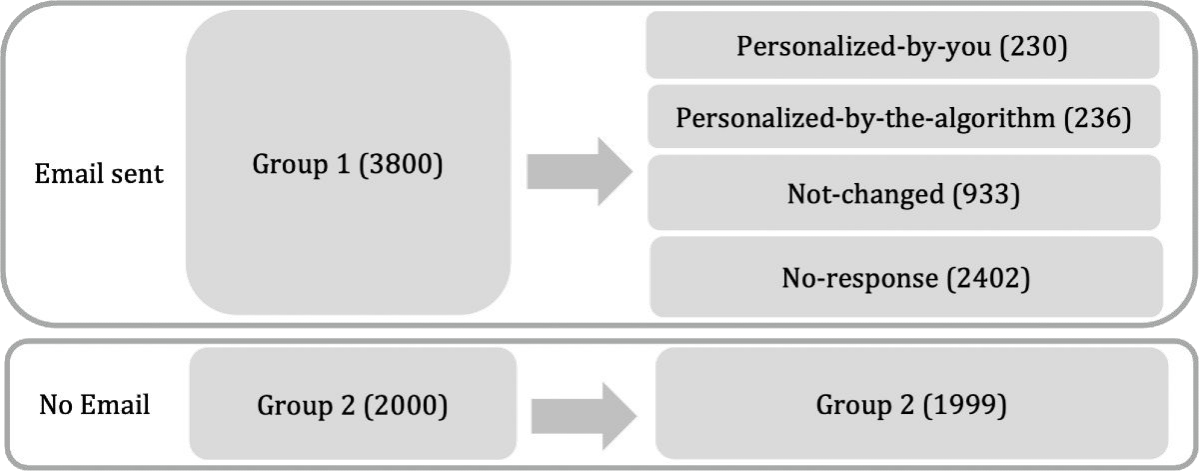
Experimental procedure.

The field study was conducted from week 8 to week 11 in 2023 (February 20 to March 19). A total of 5800 users who met our criteria were randomly chosen from the database by their user ID. In total, 3800 users were selected for group 1 and 2000 users for group 2. At the end of week 7, users in group 1 received an email containing a survey that asked whether they were satisfied with their current step goal. Users who were not satisfied, indicating the goal is “Too high” or “Too low,” could choose to personalize their goal either by themselves or through the app (see part A of Multimedia Appendix 1 for detailed information on the survey). A total of 1399 users responded, resulting in 3 subgroups: 230 users personalized their goals themselves (personalized-by-you group), 236 users had their goals personalized by the app (personalized-by-the-algorithm group), and 933 users were satisfied with their current goal and thought their current step goal was “All right” and chose to retain the default goal (not-changed group), resulting in 3 treatment groups. The remaining 2402 users did not respond to the survey (no-response group). Users in group 2 did not receive this email. The procedure is illustrated in Figure 2. Note that when scrutinizing the data, we found that 1 participant from group 2 received and completed the survey due to a technical error. Since they chose to keep the default goal, they were classified into the not-changed group, resulting in 1999 users in the no-intervention group and 933 users in the not-changed group.

In the personalized-by-you group, users set their goals within a reference range of 1000 steps to 20,000 steps. In the personalized-by-the-algorithm group, step goals were generated based on each participant’s daily step count over the 4 weeks preceding the study. Not all participants have 28 observations. For example, if users only use the app for 3 days during the previous 4 weeks before the experiment, then they only have 3 observations. As the mean steps per day do not necessarily represent a “normal day” due to outliers, we adopted a moving-window percentile rank algorithm [22,24]. Specifically, step counts were sorted in ascending order for each participant, and the observation just below the median was selected as the new step goal. For example, for a participant with 10 observations, the fourth in rank was selected, and for a participant with 7 observations, the third in rank was selected. Then, we rounded the chosen step counts to the nearest hundred as users’ step goals. We set limits on the new goal generated through this procedure. The users had to have at least 7 daily observations to generate a new goal based on their historical exercise. For those users, the algorithm constrains the goals from 2000 to 8000 steps. For example, for a user with 28 observations, if the 13th observation is over 8000, then the goal would be set as 8000. For participants with fewer than 7 observations, the goals were assigned to them based on the following rule: those who indicated that the default goal of 2000 steps was too high received a goal of 1500 steps, while those who felt it was too low were assigned 3500 steps, each for 5 days a week.

### Statistical Analysis

For all of the users in our study (N=5800), we have their data on anonymous ID, group information, step goal, age, gender, app usage duration (measured as the total number of days they have registered as a member of this app), total steps between weeks 6 and 11, and the number of days the goal was achieved during that period. To prepare the dataset for analysis, we imputed missing age values using group means, categorized missing gender as “unknown,” and replaced extreme outliers in total steps (eg, over 20 million steps per week) with the group mean for the respective week. The extreme outliers were caused by system error. An adult can only walk up to 560,000 steps a week if they walk 10 hours for 7 days based on the average walking speed. A total of 560,000 steps is far lower than 20 million steps.

A major challenge in identifying the causal effect of goal personalization on physical activity lies in the self-selection of users into different groups. The decision to personalize goals might be influenced by factors such as prior physical activity levels, app usage duration, and other individual characteristics. For instance, more active users may be more likely to engage in goal personalization. To address this issue, we utilize the propensity score matching (PSM) technique together with the difference-in-difference (DID) model, following previous research [30,31]. PSM was used to construct 3 matched control groups corresponding to the personalized-by-you, personalized-by-the-algorithm, and not-changed groups. The DID method then compares the pre- and posttreatment differences between each treatment group and its matched control group [32]. This approach helps to control the impact of exogenous and time-varying factors, such as weather, that may affect all users [32].

To ensure a sufficiently large matching pool for PSM, we combined the no-response group and group 2 (Figure 2), a total of 4401 users, as the matching group. Propensity scores were estimated using a logistic regression model based on users’ age, gender, app usage duration, total steps in weeks 6 and 7, and the number of days the step goal was achieved in those weeks. We then applied 1:1 nearest neighbor matching to construct comparable control groups for each of the 3 groups. To evaluate the quality of PSM, we checked whether the variables were balanced between each treatment group and its matched control group. The mean values of the covariates, such as the mean age and gender proportion, are closely aligned across the matched pairs, confirming the success of the PSM procedure.

To estimate the effect of goal personalization on physical activity, as measured by total steps walked per week, our DID model specification is as follows:


(1) $${\text{Steps}}_{it}=\beta_{0}+\beta_{1}{\text{Personalization}}_{it}+\theta_{i}+\lambda_{t}+\varepsilon_{it}$$

In equation 1, Steps*_it_* denotes the weekly total number of steps taken by user *i* in week *t*, and Personalization*_it_* is a binary variable indicating whether the users *i* personalized their goal in week *t*. We also included individual fixed effects (*θ_i_*) to control for unobserved heterogeneity across users and time fixed effects (*λ_t_*) to account for exogenous influence (eg, weather). We estimated the DID model 3 times, each time comparing 1 of the 3 groups with its corresponding matched control group.

The identification of the goal personalization effect using a DID model relies on the common trend assumption. To test whether the common trend assumption is met, we split the personalization effect into different weeks by including the interactions of personalization and week. The findings indicate that there is no significant difference between week 6 and week 7 across all 3 groups, supporting the common trend assumption (shown in part B of Multimedia Appendix 1).

## Results

### User Statistics

The overall mean age of the 5800 users was 54.86 years (SD 10.42), with 60% (n=5800) being women. The average app usage duration was 813.77 days (SD 536.42). Tables 1-3 present detailed characteristics of users across the personalized-by-you, personalized-by-the-algorithm, and not-changed groups and their matched control groups.

**Table 1.** Summary statistics and covariate comparison before and after matching for the personalized-by-you group.

	Personalized-by-you group	Matching group		Matched group	
	Values	Values	Mean difference^a^	Values	Mean difference^a^
Age (y), mean (SD)	52.7 (10.945)	54.786 (10.015)	–0.191	52.752 (12.225)	–0.005
Gender, n (%)					
Man	57 (24.8)	1202 (27.3)	–0.059	48 (20.9)	0.091
Unknown	8 (3.5)	674 (15.3)	–0.646	7 (3.0)	0.024
Woman	165 (71.7)	2525 (57.4)	0.319	175 (76.1)	–0.097
App usage duration (day), mean (SD)	903.596 (521.482)	780.913 (542.465)	0.235	872.213 (532.278)	0.06
Total steps in week 6, mean (SD)	48,527.896 (26,162.616)	14,648.264 (21,554.015)	1.295	47,255.104 (26,591.905)	0.049
Total steps in week 7, mean (SD)	48,593.409 (27,125.127)	13,269.404 (21,143.616)	1.302	48,968.865 (26,984.531)	–0.014
Days goal achieved in week 6, mean (SD)	5.983 (1.613)	2.094 (2.716)	2.411	5.835 (1.885)	0.092
Days goal achieved in week 7, mean (SD)	5.787 (1.820)	1.896 (2.673)	2.138	5.835 (1.642)	–0.026

a Mean difference denotes the standard mean difference.

**Table 2.** Summary statistics and covariate comparison before and after matching for the personalized-by-the-algorithm group.

	Personalized-by-the-algorithm group	Matching group		Matched group	
	Values	Values	Mean difference^a^	Values	Mean difference^a^
Age (y), mean (SD)	51.275 (11.944)	54.786 (10.015)	–0.294	50.915 (12.910)	0.030
Gender, n (%)					
Man	56 (23.7)	1202 (27.3)	–0.084	54 (22.9)	0.020
Unknown	7 (3.0)	674 (15.3)	–0.728	6 (2.5)	0.025
Woman	173 (73.3)	2525 (57.4)	0.360	176 (74.6)	–0.029
Registration period (day), mean (SD)	779.102 (503.97)	780.913 (542.465)	–0.004	796.254 (557.702)	–0.034
Total steps in week 6, mean (SD)	48,336.182 (23,668.516)	14,648.264 (21,554.015)	1.423	45,666.670 (22,468.613)	0.113
Total steps in week 7, mean (SD)	49,459.788 (24,121.172)	13,269.404 (21,143.616)	1.500	46,349.390 (23,702.009)	0.129
Days goal achieved in week 6, mean (SD)	6.093 (1.432)	2.094 (2.716)	2.793	6.114 (1.467)	–0.015
Days goal achieved in week 7, mean (SD)	6.042 (1.467)	1.896 (2.673)	2.827	6.081 (1.449)	–0.026

a Mean difference denotes the standard mean difference.

**Table 3.** Summary statistics and covariate comparison before and after matching for the not-changed group.

	Not-changed group	Matching group		Matched group	
	Values	Values	Mean difference^a^	Values	Mean difference^a^
Age (y), mean (SD)	56.656 (11.399)	54.786 (10.015)	0.164	56.329 (11.512)	0.029
Gender, n (%)					
Man	297 (31.8)	1202 (27.3)	0.097	297 (31.8)	0
Unknown	26 (2.8)	674 (15.3)	–0.761	31 (3.3)	–0.033
Woman	610 (65.4)	2525 (57.4)	0.168	605 (64.8)	0.011
Registration period (day), mean (SD)	955.374 (492.763)	780.913 (542.465)	0.354	891.212 (536.883)	0.130
Total steps in week 6, mean (SD)	39,582.826 (20,642.639)	14,648.264 (21,554.015)	1.208	40,006.05 (20,862.073)	–0.021
Total steps in week 7, mean (SD)	38,890.994 (19,347.041)	13,269.404 (21,143.616)	1.324	40,306.503 (19,527.440)	–0.073
Days goal achieved in week 6, mean (SD)	5.727 (1.586)	2.094 (2.716)	2.290	5.721 (1.634)	0.003
Days goal achieved in week 7, mean (SD)	5.755 (1.487)	1.896 (2.673)	2.595	5.827 (1.43)	–0.049

a Mean difference denotes the standard mean difference.

### Main Results

The results in Table 4 show a significant increase in physical activity among users in all 3 groups compared to their respective matched control groups. On average, users in the personalized-by-you group increased their weekly steps by 3793 (*P*<.001) from week 8 to week 11 after the personalization, compared to 2 weeks prior to the personalization. This corresponds to an average increase of 542 steps per day. Those in the personalized-by-the-algorithm group showed an increase of 4315 (*P*<.001) weekly steps (616 steps per day). These results demonstrate that both personalization strategies are effective in increasing physical activity levels. There is no significant difference between the overall effects of the 2 personalization strategies (*t*=0.354, *P*=.72). Users in the not-changed group, who did not alter their goals, also demonstrated an increase of 1759 (*P*<.001) per week (251 steps per day).

**Table 4.** The estimated effects of goal personalization on physical activity^a^.

	Personalized-by-you, steps	Personalized-by-the-algorithm, steps	Not-changed, steps
Personalization	3793.229^b^ (–1077.40)	4315.046^b^ (–994.93)	1758.642^b^ (–462.033)
Individual fixed effect	Yes	Yes	Yes
Week fixed effect	Yes	Yes	Yes
Observations	2760	2832	11,196
Individuals	460	472	1866

a Heteroskedasticity-robust standard errors are given in parentheses.

b *P*<.001.

### Heterogeneity by Prior Activity Levels

To assess the potential heterogeneity in the goal personalization effect by prior activity, we estimate specification (equation 1) on subsamples classified according to total steps walked in week 6 and week 7. Specifically, the users in each of the 2 personalization groups were further divided into 3 groups: the first quartile (low-active users), the second and third quartiles (medium-active users), and the fourth quartile (high-active users), based on their step counts prior to the study.

The results reveal significant differences in the effects of the personalized-by-you and personalized-by-the-algorithm strategies across activity levels. Among low-active users, the personalized-by-you strategy showed no significant effect (*P*=.82, see Table 5, column 1), whereas the personalized-by-the-algorithm strategy demonstrated significant effectiveness, increasing weekly total steps by 5094 from week 8 to week 11 after the personalization, compared to 2 weeks prior to the personalization (*P*=.003; see Table 5, column 4). For medium-active users, both strategies are similarly effective: personalized-by-you increased weekly steps by 5841 (*P*<.001) and personalized-by-the-algorithm by 5278 (*P*<.001) over a 4-week period after the personalization (see Table 5, columns 2 and 5). In contrast, for high-active users, personalized-by-the-algorithm was not effective (*P*=.51, see Table 5, column 6), while personalized-by-you proved to be marginally effective (*P*=.09; see Table 5, column 3).

**Table 5.** Heterogeneous effects of personalization by prior activity^a^.

	Personalized-by-you, steps			Personalized-by-the-algorithm, steps		
	Low-active users	Medium-active users	High-active users	Low-active users	Medium-active users	High-active users
Personalization	383.641 (–1694.72)	5841.661^b^ (–1476.70)	4266.063^c^ (–2598.36)	5093.521^d^ (–1766.61)	5278.006^b^ (–1476.70)	1446.37 (–2194.72)
Individual fixed effect	Yes	Yes	Yes	Yes	Yes	Yes
Week fixed effect	Yes	Yes	Yes	Yes	Yes	Yes
Observations	828	1212	720	576	1572	684
Individuals	138	202	120	96	262	114

a Heteroskedasticity-robust SEs are given in parentheses.

b *P*<.001.

c *P*<.10.

d *P*<.01.

### Robustness Check

As robustness checks, we used different matching samples for PSM. In our main study, we combined both users in the no-response group and users from group 2 as the matching sample. For robustness checks, we treated these 2 groups separately as distinct matching samples. We then reestimated the same DID models. The results remain robust, with all 3 groups (personalized-by-you, personalized-by-the-algorithm, and not-changed groups) showing significant effects (shown in parts C and D in Multimedia Appendix 1).

## Discussion

### Principal Results

Given the potential benefits of goal personalization on physical activity and the lack of solid evidence in previous literature, a large-scale field study was conducted to examine the potential of personalization in prompting health behavior. Our findings demonstrate that goal personalization effectively increased physical activity, resulting in an average increase of over 500 daily steps over a 4-week period. This increase is meaningful at the individual level, as prior meta-analytic evidence based on over 200,000 participants indicates that an additional 500 steps per day is associated with a 7% reduction in cardiovascular-related mortality [33]. Our findings also demonstrate that even participants who only completed the survey but did not change their goal increased their weekly steps by 1759 over a 4-week period. The increase may be attributed to a reminder effect from receiving the email and completing the survey [34,35]. However, post hoc analyses reveal that users in both goal personalization groups take significantly more steps than those in the not-changed groups. This suggests that the observed increase in steps is not solely due to the reminder effect. Specifically, compared with the unchanged group, the users in the personalized-by-you group showed a marginally significant increase (*P*=.09), while the users in the personalized-by-the-algorithm group showed a significant increase (*P*=.02).

Moreover, this study also compared the effectiveness of 2 personalization strategies. While the overall effects of these 2 strategies are similar, heterogeneity tests based on prior activity levels indicate varying effectiveness across distinct groups. The personalized-by-you strategy proves effective for medium and high-active users, whereas the personalized-by-the-algorithm significantly boosts total steps for low-active and medium-active users.

We argue the potential reason why personalized-by-the-algorithm is effective for low-active users, while personalized-by-you is not, is as follows. Low-active users may overestimate their physical abilities when personalizing the goal by themselves, leading to goals that are less realistic and motivating. In contrast, goals generated by the algorithm are based on users’ previous exercise data, likely aligning better with their capabilities. This alignment enhances individuals’ self-accountability and competence and drives better health outcomes [36]. A post hoc analysis shows that low-active users in the personalized-by-you group set higher goals than those in the personalized-by-the-algorithm group—3730 steps versus 2844 steps—a difference that is marginally significant (*P*=.07). This finding underscores the importance of personalized-by-the-algorithm in setting realistic and motivating goals for low-active users.

The reason why personalized-by-you is effective for highly active users, while personalized-by-the-algorithm is not, is as follows. High-active users, being more experienced with exercise, may be capable of setting goals that better align with their ability, situation, and standard than the algorithm. Further analysis supports this, showing that high-active users in the personalized-by-you group set lower, more realistic goals (5700 steps on average) compared to those in the personalized-by-the-algorithm group (7061 steps on average; *P*=.002). The higher goals set by the algorithm might be too challenging and therefore demotivating these users.

These findings advance goal-setting theory in 3 ways. First, prior research provides mixed guidance on goal type: while the SMART framework emphasizes specific and attainable goals [12,13], other work stresses specific and challenging goals [14,15]. Both approaches have limitations: too-easy goals may fail to motivate meaningful health behavior. This study indeed shows that the default 2000-step goal was less motivating than the average 4747-step goal set in the personalized groups. Conversely, difficult goals can discourage users [11,14], as this study shows that the average 7061-step goals for high-active users in the personalized-by-the-algorithm group only had a similar effect as the 2000-step goal. Our study highlights the value of personalized goals that align with individuals’ abilities, characteristics, and contexts. Second, beyond goal type, the study contributes to how to best set the goals by identifying 2 distinct goal personalization strategies. Third, the study examines the moderating role of prior activity level, offering theoretical insights into the conditions under which different personalization strategies are most effective. In sum, the findings extend goal-setting theory by clarifying what types of goals should be set, how goals can be personalized (methods), and when different personalization strategies are most effective (boundary conditions).

As for practical contribution, given the findings, mHealth technology companies should prioritize algorithmic personalization or, at the very least, frame the choices of personalization in a manner that encourages low-active users to opt for algorithm-based personalization. However, for high-active users, it is important to give them the opportunity to personalize their goals by themselves, as they are more motivated by self-control, autonomy, and self-accountability and are better equipped to set goals that align with their abilities.

### Limitations

This research has limitations that warrant consideration. First, the participants were not randomly assigned to different treatment groups but made the decision by themselves, which could introduce potential biases. Although we employ PSM and DID to address the issue, only a randomized field experiment can fully mitigate such biases. Second, the lack of postexperiment data prevents us from assessing the long-term sustainability of the goal personalization effect. Third, we do not have data to test whether the goal personalization effect extends to other health-related activities, such as gym attendance or weight management, which limits the generalizability of our findings. Fourth, this study compares personalized goals to a fixed 2000-step goal, without considering other types of goals (eg, 5000-step goal), which may constrain the scope of the comparison. Finally, there are other goal personalization algorithms, such as machine learning approaches that predict step goals using previous step data and goal histories [23], or simple goal adjustment approaches based on goal achievement [37,38]. Future studies can investigate the comparative effectiveness of these goal personalization methods. Furthermore, more intelligent algorithms that incorporate contextual factors—such as users’ moods and weather conditions—should be developed and empirically tested in future research to examine whether they can generate more motivating personalized goals and encourage users to be more physically active.

### Conclusions

This study investigated the effects of 2 goal personalization strategies on increasing users’ physical activity levels through an open-access mHealth app. The results show that both strategies significantly promote physical activity—by over 500 additional steps per day—with improvements that are meaningful at the individual level, particularly in reducing health risks. Moreover, the study identified the applicability of each personalization strategy: the personalized-by-you approach is more effective for medium- and high-active users, while the personalized-by-the-algorithm strategy works better for low- and medium-active users. These insights can inform the design of the goal management features in mHealth apps to enhance the effectiveness of health interventions.

## Supplementary material

10.2196/81779 Multimedia Appendix 1 Survey instruments, goal personalization effects, and robustness checks across sample variations.

## Abbreviations

DID: difference-in-difference
mHealth: mobile health
PSM: propensity score matching
SMART: specific, measurable, achievable, realistic, time-bound
