# Supplementary material for: Effectiveness of Step Goal Personalization Strategies on Physical Activity in a Mobile Health App: A Field Study
Source: JMIR Mhealth Uhealth. 2026 Feb 18;14:e81779. doi: 10.2196/81779 (PMC12916092; doi:10.2196/81779)
Supplement: Multimedia Appendix 1 [file mhealth-v14-e81779-s001.docx]

**Multimedia Appendix 1**

**Appendix A: The survey** 2

**Appendix B: Goal personalization effect in different weeks** 3

**Appendix C: Robustness check: Users not responding to the survey as the matching sample** 4

**Appendix D: Robustness check: Users in the original control group as the matching sample** 5

**Appendix A: The survey**

Introduction: Steps are a convenient way to measure how much you walk. It is healthy to exercise every day. Your current goal is to take at least 2000 steps 5 days a week. We would like to hear from you what you think of your steps goal and whether you want to set a different goal for yourself.

**1. What do you think of your step goal?** (A good goal motivates you to take an extra step and is realistic)

⭘ Too low ⭘ All right (to question 4) ⭘ Too high

**2. Which step goal do you want?**

I prefer:

⭘ I like to set my step goal myself (to question 4)

⭘ I would like the app to calculate a step goal based on my current exercise pattern (skip question 4)

**3. What daily step goal do you set for yourself at least 5 days a week?**

Minimum 1,000 steps, maximum 20,000 steps: **__________________**

We're curious to hear your thoughts on the components of your weekly goal. The last few questions are about this.

**4. In your opinion, what works best for you to achieve your exercise goals?**

⭘ Only a steps goal

⭘ Only an exercise goal in minutes

⭘ A step goal and an exercise goal in minutes

Thanks for sharing. Together we make SamenGezond better! If you have set a step goal in this questionnaire, you can expect it incorporated in your weekly goal next week.

**Appendix B: Goal personalization effect in different weeks**

Table 1. The Estimated Effects of Goal Personalization on Physical Activity in different weeks

|  | Personalized-by-you | | Personalized-by-the-algorithm | | Not-changed | |
| --- | --- | --- | --- | --- | --- | --- |
| Personalization | 3,793.229*** |  | 4,315.046*** |  | 1,758.642*** |  |
|  | (1,077.40) |  | (-994.93) |  | (-462.033) |  |
| Personalize_week7 |  | -1,648.25 |  | 440.886 |  | -992.285 |
|  |  | (-1,770.97) |  | (-1,634.87) |  | (-743.948) |
| Personalize_week8 |  | 1,781.30 |  | 5,662.826*** |  | 1,419.193+ |
|  |  | (-1,687.40) |  | (-1,604.63) |  | (-748.513) |
| Personalize_week9 |  | 1,741.74 |  | 4,883.894** |  | 985.595 |
|  |  | (-1,760.39) |  | (-1,625.20) |  | (-779.306) |
| Personalize_week10 |  | 2,898.661+ |  | 3,577.178* |  | 635.62 |
|  |  | (-1,714.80) |  | (-1,573.10) |  | (-763.431) |
| Personalize_week11 |  | 5,454.717** |  | 4,018.055* |  | 2,009.591* |
|  |  | (-1,956.74) |  | (-1,791.63) |  | (-826.933) |
|  |  |  |  |  |  |  |
| Individual fixed effect | YES | YES | YES | YES | YES | YES |
| Week fixed effect | YES | YES | YES | YES | YES | YES |
| Observations | 2,760 | 2,760 | 2,832 | 2,832 | 11,196 | 11,196 |
| Individuals | 472 | 472 | 460 | 460 | 1,866 | 1,866 |

Note: + p<0.1; * p<0.05; ** p<0.01; *** p<0.001; Heteroskedasticity-robust (HS) Robust standard errors in parenthesis

**Appendix C: Robustness check: Users not responding to the survey as the matching sample**

Table 2. The Estimated Effects of Goal Personalization on Physical Activity

|  | Personalized-by-you | Personalized-by-the-algorithm | Not-changed |
| --- | --- | --- | --- |
| Personalization | 5,568.053*** | 5,147.273*** | 2,702.445*** |
|  | (1,037.088) | (1,108.132) | (494.871) |
| Individual fixed effect | YES | YES | YES |
| Week fixed effect | YES | YES | YES |
| Observations | 2,760 | 2,832 | 11,196 |
| Individuals | 472 | 460 | 1,866 |

Note: + p<0.1; * p<0.05; ** p<0.01; *** p<0.001; HS Robust standard errors in parenthesis

**Appendix D: Robustness check: Users in the original control group as the matching sample**

Table 3 The Estimated Effects of Goal Personalization on Physical Activity

|  | Personalized-by-you | Personalized-by-the-algorithm | Not-changed |
| --- | --- | --- | --- |
| Personalization | 2,920.922** | 1,865.458+ | 1,673.952*** |
|  | (981.450) | (1,033.103) | (471.495) |
| Individual fixed effect | YES | YES | YES |
| Week fixed effect | YES | YES | YES |
| Observations | 2,760 | 2,832 | 11,196 |
| Individuals | 472 | 460 | 1,866 |

Note: + p<0.1; * p<0.05; ** p<0.01; *** p<0.001; HS Robust standard errors in parenthesis

# Appendix E: Figure 1 Screenshots of the (Dutch-language) mobile health app


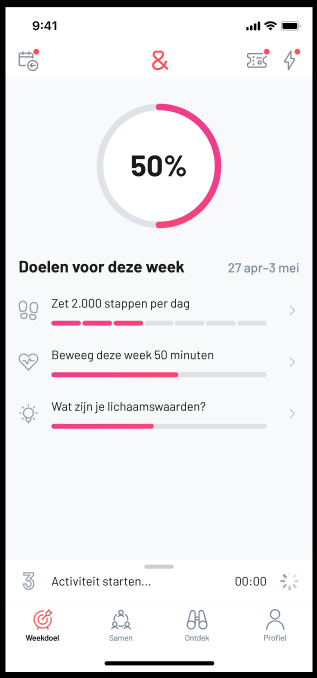

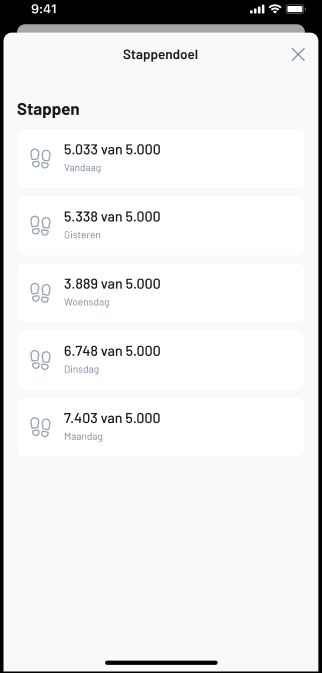


**Appendix F: Figure 2 Experimental Procedure**

Email sent

No Email

Personalized-by-you (230)

Personalized-by-the-algorithm (236)

Not-changed (933)

No-response (2,402)

Group 2 (1999)

Group 2 (2000)

Group 1 (3,800)
